# Adherence to Breast Cancer Screening Guidelines Among Age‐Eligible US Women: Findings From NHIS 2021

**DOI:** 10.1002/cam4.71423

**Published:** 2025-12-12

**Authors:** Monalisa Chandra, Joël Fokom Domgue, Robert Yu, Sanjay Shete

**Affiliations:** ^1^ Department of Epidemiology The University of Texas MD Anderson Cancer Center Houston Texas USA; ^2^ Division of Cancer Prevention and Population Sciences The University of Texas MD Anderson Cancer Center Houston Texas USA; ^3^ Department of Biostatistics The University of Texas MD Anderson Cancer Center Houston Texas USA

**Keywords:** adherence, breast cancer, cancer prevention, health, health disparities, mammogram, screening, screening guidelines, smoking, women's health

## Abstract

**Background:**

Despite the proven benefits of early breast cancer detection in reducing mortality, adherence to breast cancer screening guidelines in the United States is suboptimal. In this study, we assessed the prevalence and factors associated with adherence to breast cancer screening guidelines.

**Methods:**

Using nationally representative data from the National Health Interview Survey 2021, we included breast cancer screening‐eligible women (aged 50–74). Descriptive statistics and population‐weighted multivariable logistic regression were employed to examine breast cancer screening adherence per the United States Preventive Services Task Force guidelines, and its determinants in this population.

**Results:**

Of the 6814 screening‐eligible women included in this study, 76.4% adhered to breast cancer screening guidelines. Asians (adjusted odds ratio [AOR]: 0.59 (95% confidence interval [95% CI]: 0.42–0.83), *p* = 0.003), current cigarette smokers (AOR: 0.62 (95% CI: 0.50–0.76), *p* < 0.0001), individuals with a precollege educational attainment (less than high school [AOR: 0.60 (95% CI: 0.45–0.80), *p* = 0.0005] and high school or GED [AOR: 0.79 (95% CI: 0.66–0.94), *p* < 0.001]), those who reported a poor/fair health status (AOR: 0.71 (95% CI: 0.58–0.86), *p* = 0.0006), single/widowed/separated/divorced (AOR: 0.71 (95% CI: 0.61–0.82), *p* < 0.0001), and uninsured (AOR: 0.24 (95% CI: 0.18–0.32), *p* < 0.0001) women had lower odds of being adherent to breast cancer screening. Non‐Hispanic Blacks (AOR: 1.81 (95% CI: 1.38–2.36), *p* < 0.0001), women who had a routine checkup in the past 2 years (AOR: 11.24 (95% CI: 7.31–17.29), *p* < 0.0001), and those with a personal cancer history (AOR: 1.44 (95% CI: 1.18–1.76), *p* = 0.0003) had higher odds of being adherent to breast cancer screening guidelines.

**Conclusion:**

Adherence to breast cancer screening in the United States remained below the Healthy People 2030 goal, with important variations across sociodemographic, behavioral, and health‐related factors. Public health actions such as integrating breast cancer awareness activities into smoking cessation programs, encouraging and equipping healthcare professionals to use culturally tailored interventions, and reinforcing community education about breast cancer and its prevention will help increase breast cancer screening adherence among US women.

## 1 Introduction

Breast cancer is the second most common cancer among women in the United States, accounting for 30% of all new female cancers every year, and the second leading cause of cancer mortality [1]. In addition to demographic factors such as race, ethnicity, and insurance [2], breast cancer risk is associated with nonmodifiable factors (such as genetic mutation, older age, early menarche, late menopause, and dense breast tissue) and modifiable risk factors (such as lack of physical activity, obesity, late pregnancy, alcohol consumption, and tobacco use) [3, 4]. In the past decade, breast cancer incidence rates have increased in the United States, especially among women aged 20–49 years [5]. This is partly due to the growing prevalence of risk factors for breast cancer in the general population, combined with a stable rate of screening in the target population over the years [6, 7]. Although breast cancer mortality has decreased across all racial groups, non‐Hispanic Black (NHB) women are still disproportionately affected, with a 40% higher mortality rate compared to non‐Hispanic Whites (NHW) [8].

Screening mammograms help detect breast cancer at an early stage, which reduces the need for invasive treatments and decreases breast cancer–related mortality [9, 10]. The United States Preventive Services Task Force (USPSTF) guidelines recommended until 2023 that women aged 50–74 should get screened for breast cancer every other year [11]. Screening mammogram uptake varies across different groups in the United States, with the lowest rates being found among American Indians or Alaskan Natives [12], those with educational attainment less than high school, those below 200% of the federal poverty level [13], those without health insurance, and those without a primary healthcare provider [12]. Additional attributes of breast cancer screening included marital status and health beliefs [14]. However, there is a dearth of information on breast cancer screening adherence in certain high‐risk groups such as tobacco smokers, sexual minorities, and cancer survivors.

Smoking tobacco is the leading modifiable factor of cancer and accounts for 30% of cancer deaths in the United States [15]. In addition to a causal link between lifelong smoking exposure and breast cancer risk [4], smoking tobacco was found to be associated with p53 mutation in human breasts [16] and to strengthen the association between polycyclic aromatic hydrocarbons and breast cancer risks [17]. Breast cancer was also associated with duration, intensity, cumulative exposure, and latency of cigarette smoking [18]. Considering the increased breast cancer risk associated with smoking, adherence to routine screening mammograms may benefit women who are current or former smokers. Breast cancer screening adherence is essential for cancer survivors who are at a high risk of cancer recurrence, new primary cancer, or late effects of previous treatments [19]. Breast cancer risks are also high among sexual minorities, who are often identified with risky health behaviors (smoking and drinking) [20], limited access to healthcare [21, 22], which results in poorer health outcomes [23, 24]. Despite known risks, limited information is available on breast cancer screening adherence in this population. To address these gaps, this study aimed to examine demographic and behavioral differences in adherence to breast cancer screening guidelines among US women.

## 2 Methods

### 2.1 Study Population and Data Collection

This study used National Health Information Survey (NHIS) 2021 data, which is administered annually to monitor the health of the noninstitutionalized US population by collecting and analyzing demographic and health‐related information. NHIS 2021 data were collected between January 2021 and December 2021 using computer‐assisted or face‐to‐face interviews with an adult household member. All respondents provided informed consent before participation, and the National Center for Health Statistics research ethics review board approved the survey [25]. Our study followed the Strengthening the Reporting of Observational Studies in Epidemiology (STROBE) guidelines and included 6814 women aged 50–74, selected from a sample of 29,482 adults. The current study was exempted from ethics review because we used deidentified publicly available data.

### 2.2 Measures

#### 2.2.1 Outcome Variable

##### 2.2.1.1 Breast Cancer Screening Adherence

Adherence to breast cancer screening was defined according to the USPSTF guidelines that were in effect at the time of the survey [11, 26], and measured using the two nested questions, “Have you ever had a mammogram?” and “About how long has it been since your most recent mammogram?” Respondents were considered as adherent if they had a mammogram within the past 2 years, and not adherent if they had never had a mammogram or had a mammogram more than 2 years ago.

### 2.3 Independent Variables

Cigarette smoking, personal cancer history, routine checkups, perceived health condition, and demographic characteristics were the independent variables of interest. Cigarette smoking was measured using the questions “Have you smoked at least 100 cigarettes in your entire life?” and “Do you now smoke cigarettes every day, some days, or not at all?” We recoded the NHIS smoking status categories [27] into current smokers (a combination of current everyday smokers and current someday smokers), former smokers, never smokers, and others (a combination of smoker status unknown and unknown if ever smoked). Information on personal cancer history was also included using the question, “Have you ever been told by a doctor or other health professional that you had … Cancer or a malignancy of any kind?” The response options were “yes” and “no.”

Routine checkups were measured using the question, “About how long has it been since you last saw a doctor or other health professional for a wellness visit, physical, or general purpose checkup?” The response options included (1) “never,” (2) “within past year (anytime less than 12 months ago)”, (3) “within last 2 years (1 year but less than 2 years ago),” (4) “within the last 3 years (2 years but less than 3 years ago),”, (5) “within the last 5 years (3 years but less than 5 years ago),” (6) “within the last 10 years (5 years but less than 10 years ago),” (7) “10 years ago or more.” Those who chose options (2) and (3) were recoded as “checked less than 2 years ago,” and those who chose options (1), (4), (5), (6), and (7) were recoded as “Never or more than 2 years ago.” Perceived health condition was measured using the question, “Would you say your health in general is excellent, very good, good, fair, or poor?” The responses were recoded as “fair/poor” and “excellent/very good/good.”

Other independent variables included were sociodemographic characteristics. They were categorizes as follows: age groups (50–64/65–74), race or ethnicity (non‐Hispanic White/non‐Hispanic Black/Hispanic/non‐Hispanic Asian/non‐Hispanic others [including Alaskan Natives, American Indians, Native Hawaiian, other Pacific Islanders, some other race, and multiple races]), marital status (married or living as married/single, widowed, separated, divorced), education attainment level (less than high school/high school or General Educational Development (GED) test/some college/bachelor's or higher), poverty ratio (< 2, ≥ 2), urbanicity (metropolitan [large central metro, large fringe metro, medium and small metro]/nonmetropolitan [micropolitan and noncore]), born in the United States (Yes/No), have health insurance (Yes/No), and sexual orientation (straight/gay or lesbian/bisexual/something else/don't know). The poverty ratio was defined as the ratio of family income to the poverty threshold. The poverty threshold was obtained from the Census Bureau for the previous calendar year. A poverty ratio equal to or more than two implies an annual household income at or above 200% of the federal poverty threshold [28]. Urbanicity was categorized based on the 2010 Office of Management and Budget metropolitan and nonmetropolitan classification of counties [29]. The sexual orientation variable was defined based on the question “Do you think of yourself as gay/lesbian; straight, that is, not gay/lesbian; bisexual; something else; or you don't know the answer?”

### 2.4 Statistical Analysis

Analyses were conducted using SAS survey procedures (version 9.4; SAS Institute). The NHIS developers calculated specific weights for the 2021 data sets to reflect the likelihood of being selected in the survey and to account for nonresponse adjustment among the selected sample. In addition to weighting, to account for the stratified cluster sampling design of the NHIS, key nesting variables were created to accurately estimate the sampling error. Frequencies, weighted percentages with 95% confidence intervals, and cross‐tabulation of independent variables by the outcome variable were calculated using PROC SURVEYFREQ with weight, strata, and cluster. We evaluated group differences in breast cancer screening adherence stratified by the independent variables using the Wald chi‐square test. Continuous variables were analyzed using the SURVEYMEANS procedure. We conducted multivariable regression using the SURVEYLOGISTIC function which accounts for weights, strata, and clusters to estimate the adjusted odds ratio with a 95% confidence interval. Statistical tests were two‐tailed, and *p* < 0.05 was considered statistically significant.

## 3 Results

The study population included 6814 women aged between 50 and 74 living in the United States. Sixty‐four percent (64.4%) of the study population were aged 50–64 years, 71.5% were NHWs, 62.6% were married or living as married, 43.3% had a tertiary level educational attainment, 95% had an income to poverty ratio greater than 2, 84.7% were metropolitan residents, 79.4% were US‐born, 94.7% had health insurance, 82.9% had routine checkup in the last 2 years, 81.5% reported good/very good/excellent perceived health condition, 15.4% had a personal cancer history, 12.1% were current smokers, and 96.9% identified as straight (Table 1).

**TABLE 1:** Characteristics of the study population (US women aged 50–74 years) according to breast cancer screening adherence—National Household Information Survey 2021.

Characteristics	Categories^a^	Overall		Adherent			Not adherent			
		*N*	wt.%	wt.%	95% CI^b^		wt.%	95% CI		*p*
		6814	100							
Had a mammogram within the past 2 years	Yes	5200	76.4							
	No	1614	23.6							
Age (in years)	50–64	4080	64.4	75.9	74.5	77.4	24.1	22.6	25.5	0.298
	65–74	2734	35.6	77.2	75.3	79.0	22.8	21.0	24.7	
Race or ethnicity	Non‐Hispanic White	4827	71.5	76.6	75.2	77.9	23.4	22.1	24.8	< 0.0001
	Non‐Hispanic Black	824	12.5	82.6	79.4	85.7	17.4	14.3	20.6	
	Hispanic	434	8.0	71.4	66.4	76.4	28.6	23.6	33.6	
	Non‐Hispanic Asian	349	5.8	68.0	62.4	73.6	32.0	26.4	37.6	
	Non‐Hispanic others	146	2.2	66.9	58.1	75.6	33.1	24.4	41.9	
Marital status	Living as married or married	3459	62.6	78.6	77.1	80.1	21.4	19.9	22.9	< 0.0001
	Single, widowed, separated, divorced	3355	37.4	72.6	70.8	74.4	27.4	25.6	29.2	
Educational attainment	Less than high school	571	11.6	64.9	60.1	69.8	35.1	30.2	39.9	< 0.0001
	High school/GED	1634	33.2	73.5	71.1	75.9	26.5	24.1	28.9	
	Some college	735	11.9	81.1	77.8	84.4	18.9	15.6	22.2	
	Bachelor's and higher degree (tertiary)	2515	43.3	81.0	79.3	82.7	19.0	17.3	20.7	
Poverty ratio^c^	< 2	381	5.0	67.4	61.5	73.3	32.6	26.7	38.5	0.001
	≥ 2	6433	95.0	76.8	75.6	78.1	23.2	21.9	24.4	
Urbanicity^d^	Metropolitan	5730	84.7	76.9	75.6	78.2	23.1	21.8	24.4	0.0115
	Nonmetropolitan	1084	15.3	73.3	70.8	75.9	26.7	24.1	29.2	
Born in the United States	Yes	5611	79.4	76.8	75.6	78.1	23.2	21.9	24.4	0.013
	No	1014	17.4	72.9	69.7	76.2	27.1	23.8	30.3	
	Missing	189	3.1	83.9	77.1	90.6	16.1	9.4	22.9	
Health insurance	No	308	5.3	43.4	36.8	50.0	56.6	50.0	63.2	< 0.0001
	Yes	6492	94.7	78.3	77.1	79.4	21.7	20.6	22.9	
Routine checkup	Never or more than 2 years ago	224	17.1	25.5	18.3	32.7	74.5	67.3	81.7	< 0.0001
	Checked less than 2 years ago	1106	82.9	82.0	79.6	84.4	18.0	15.6	20.4	
Perceived health condition	Excellent/very good/good	5555	81.5	78.0	76.7	79.3	22.0	20.7	23.3	< 0.0001
	Fair/poor	1257	18.5	69.1	66.0	72.3	30.9	27.7	34.0	
Personal cancer history	Yes	1157	15.4	82.2	79.7	84.6	17.8	15.4	20.3	< 0.0001
	No	5646	84.6	75.3	74.0	76.6	24.7	23.4	26.0	
Smoking status	Current smoker	856	12.1	63.7	60.0	67.4	36.3	32.6	40.0	< 0.0001
	Former smoker	1725	23.7	77.1	74.8	79.4	22.9	20.6	25.2	
	Never smoker	4056	61.3	78.1	76.7	79.6	21.9	20.4	23.3	
	Unknown	177	2.8	86.9	80.3	93.4	13.1	6.6	19.7	
Sexual orientation	Straight	6355	96.9	76.4	75.2	77.6	23.6	22.4	24.8	0.0142
	Gay or lesbian	101	1.4	77.9	69.3	86.5	22.1	13.5	30.7	
	Bisexual	49	0.6	61.7	47.6	75.7	38.3	24.3	52.4	
	Something else	25	0.4	83.8	70.0	97.6	16.2	2.4	30.0	
	Don't know	52	0.7	59.4	43.7	75.1	40.6	24.9	56.3	

^a^ Some of the variable categories do not add up to 6814 because of missing responses.

^b^ Confidence interval.

^c^ Ratio of family income to poverty threshold. The poverty threshold was obtained from the Census Bureau for the previous calendar year.

^d^ Metropolitan includes geographical areas defined as large central metro, large fringe metro, medium, and small metro. Nonmetropolitan includes micropolitan and noncore.

**FIGURE 1:**
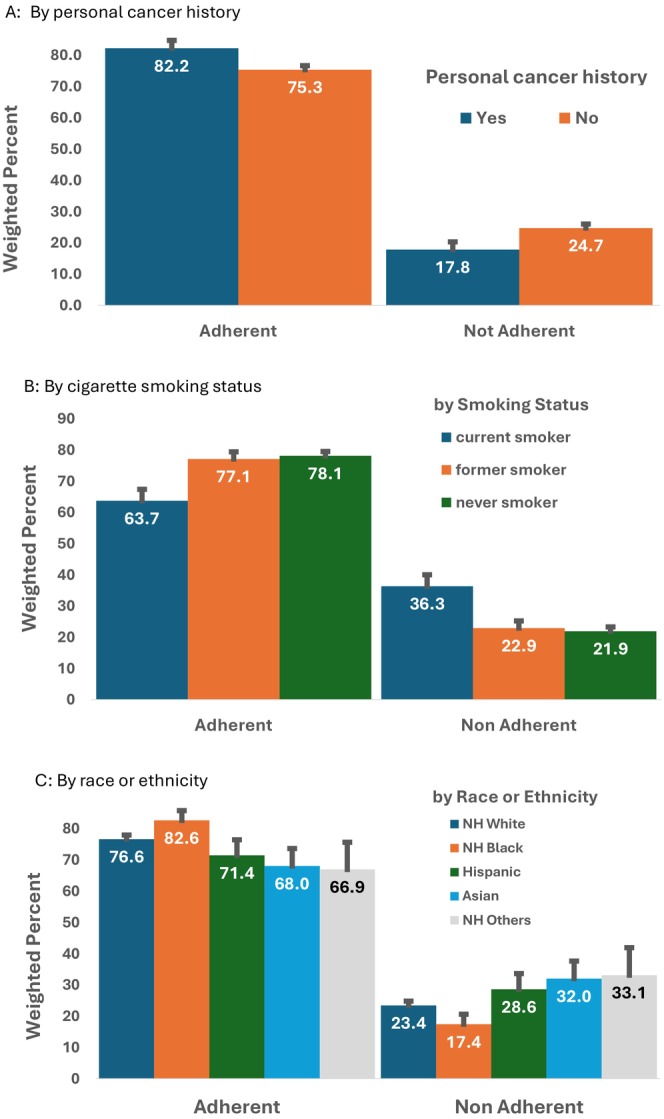
Adherence to breast cancer screening among US women aged 50–74 years, by personal cancer history, (A), cigarette smoking (B), and race/ethnicity (C), NHIS 2021. (A) By personal cancer history: Personal cancer history was measured by asking “Have you EVER been told by a doctor or other health professional that you had …Cancer or a malignancy of any kind?” The available responses were “yes” and “no.” (B) By cigarette smoking status: The current smokers are a combination of individuals who are current everyday smokers and current someday smokers. The other categories include never smokers, and others (a combination of individuals whose smoker status unknown and it is unknown if they ever smoked). (C) By race or ethnicity. The race or ethnicity categories are defined as NH White: Non‐Hispanic Whites; NH Black: Non‐Hispanic Black; Hispanic; Asian‐ Non‐Hispanic Asians; NH others: this group includes Alaskan Natives, American Indians, Native Hawaiian, other Pacific Islanders, some other race and multiple races.

Overall, 76.4% of US women adhered to breast cancer screening guidelines. In the stratified analysis, we found that individuals with a personal cancer history (82.2%), never smokers (78.1%), and NHBs (82.6%) had higher rates of adherence to breast cancer screening guidelines than those without a personal cancer history, current or former smokers, and NHWs, respectively (Figure 1A–C). In addition, 64.9% of women with educational attainment less than high school had lower rates of adherence to breast cancer screening guidelines than those with higher educational attainment (Table 1).

**FIGURE 2:**
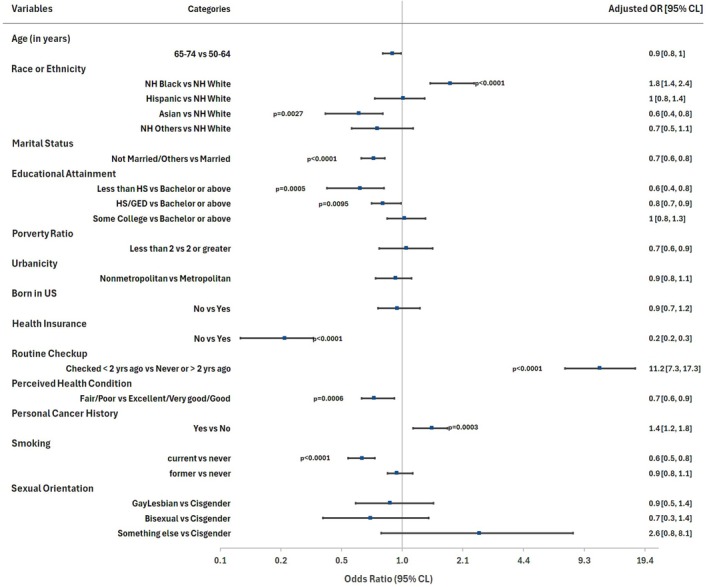
Illustrative Forest plots representing multivariable logistic regression of factors associated with breast cancer screening adherence among US women aged 50–74 years, NHIS 2021. The race or ethnicity categories are defined as NH White: Non‐Hispanic Whites; NH Black: Non‐Hispanic Black; Hispanic; Asian‐ Non‐Hispanic Asians; NH others: this group includes Alaskan Natives, American Indians, Native Hawaiian, other Pacific Islanders, some other race and multiple races. Note: only *p*‐values lower than 0.05 are presented in the Figure.

In the multivariable analysis (Table 2 and Figure 2), non‐Hispanic Asians had 41% lower odds (AOR: 0.59; 95% CI: 0.42–0.83; *p* = 0.003) of being breast cancer screening adherent than NHWs. Current smokers had 38% lower odds (AOR: 0.62; 95% CI: 0.50–0.76; *p* < 0.0001) of being breast cancer screening adherent than never smokers. Women with educational attainment below high school had 40% lower odds (AOR: 0.60; 95% CI: 0.45–0.80; *p* = 0.0005), and those with high school or GED had 21% lower odds (AOR: 0.79; 95% CI: 0.66–0.94; *p* < 0.001) of being breast cancer adherent than those with tertiary level of educational attainment. Single, widowed, separated, or divorced women had 29% lower odds (AOR: 0.71; 95% CI: 0.61–0.82; *p* < 0.0001) of being breast cancer screening adherent than women who were married or living as married. Women without health insurance had 76% lower odds (AOR: 0.24; 95% CI: 0.18–0.32; *p* < 0.0001) of being breast cancer screening adherent than those with health insurance. Women who reported poor/fair health status had 29% lower odds (AOR: 0.71; 95% CI: 0.58–0.86; *p* = 0.0006) of being breast cancer screening adherent than those who reported excellent/very good/good health condition. NHBs had 81% higher odds (AOR: 1.81; 95% CI: 1.38–2.36; *p* < 0.0001) of being breast cancer screening adherent than NHWs. Individuals with personal cancer history had 44% higher odds (AOR: 1.44; 95% CI: 1.18–1.76; *p* = 0.0003) of being breast cancer screening adherent than women without personal cancer history. Women who had a routine checkup exam within the last 2 years had 1024% higher odds (AOR: 11.24; 95% CI: 7.31–17.29; *p* < 0.0001) of being breast cancer screening adherent than those who never had a routine checkup or had it more than 2 years ago. No significant difference in breast cancer screening adherence was found based on age, poverty ratio, urbanicity, place of birth, and sexual orientation (Table 2 and Figure 2).

**TABLE 2:** Multivariable survey‐weighted logistic regression analyses of factors associated with breast cancer screening adherence among US women aged 50–74 years: National Health Information Survey 2021.

Characteristics	Category^a^	AOR^b^	Lower 95% CI	Upper 95% CI	*p*
Age (in years)	65–74	0.89	0.78	1.03	0.093
	50–64	Ref.	Ref.	Ref.	Ref.
Race or ethnicity	Non‐Hispanic Black	1.81	1.38	2.36	< 0.0001
	Hispanic	1.01	0.76	1.36	0.923
	Non‐Hispanic Asian	0.59	0.42	0.83	0.003
	Non‐Hispanic others	0.74	0.49	1.12	0.15
	Non‐Hispanic White	Ref.	Ref.	Ref.	Ref.
Marital status	Single, widowed, separated, divorced	0.71	0.61	0.82	< 0.0001
	Living as married or married	Ref.	Ref.	Ref.	Ref.
Educational attainment	Less than high school	0.60	0.45	0.80	0.0005
	High school/GED	0.79	0.66	0.94	< 0.001
	Some college	1.03	0.81	1.31	0.804
	Bachelor's and higher degree (tertiary)	Ref.	Ref.	Ref.	Ref.
Poverty ratio^c^	< 2	1.05	0.77	1.44	0.74
	≥ 2	Ref.	Ref.	Ref.	Ref.
Urbanicity^d^	Nonmetropolitan	0.92	0.77	1.10	0.37
	Metropolitan	Ref.	Ref.	Ref.	Ref.
Born in the United States	No	0.94	0.74	1.20	0.636
	Yes	Ref.	Ref.	Ref.	Ref.
Health insurance	No	0.24	0.18	0.32	< 0.0001
	Yes	Ref.	Ref.	Ref.	Ref.
Routine checkup	Checked less than 2 years ago	11.24	7.31	17.29	< 0.0001
	Never or more than 2 years ago	Ref.	Ref.	Ref.	Ref.
Perceived health condition	Fair/poor	0.71	0.58	0.86	0.0006
	Excellent/very good/good	Ref.	Ref.	Ref.	Ref.
Personal cancer history	Yes	1.44	1.18	1.76	0.0003
	No	Ref.	Ref.	Ref.	Ref.
Smoking status	Current smoker	0.62	0.50	0.76	< 0.0001
	Former smoker	0.94	0.79	1.11	0.434
	Never smokers	Ref.	Ref.	Ref.	Ref.
Sexual orientation	Gay or lesbian	0.87	0.52	1.44	0.577
	Bisexual	0.68	0.34	1.36	0.273
	Something else	2.57	0.82	8.07	0.105
	Don't know	0.58	0.26	1.30	0.186
	Straight	Ref.	Ref.	Ref.	Ref.

*Note:* The reference category for the outcome variable in this model is nonadherence to breast cancer screening guidelines, defined as individuals who report not having had a mammogram in the last 2 years.

Abbreviation: CI, confidence interval.

^a^ Some of the variable categories do not add up to 6814 because of missing responses.

^b^ Adjusted odds ratio.

^c^ Ratio of family income to poverty threshold. The poverty threshold was obtained from the Census Bureau for the previous calendar year.

^d^ Metropolitan includes geographical areas defined as large central metro, large fringe metro, medium, and small metro. Nonmetropolitan includes micropolitan and noncore.

To compare the characteristics of women who never had a mammogram and those who had been screened more than 2 years ago, we divided the nonadherent category into two groups (see Table S1).

## 4 Discussion

Using nationally representative data, we found that breast cancer screening adherence was associated with certain behavioral and sociodemographic characteristics. While Asian women, current smokers, those with lower levels of educational attainment, those who are single/widowed/separated/divorced, and uninsured women had lower odds of breast cancer screening adherence, NHBs and cancer survivors had higher odds of being adherent to breast cancer screening.

Our finding of lower odds of mammogram adherence among Asians is supported by previous reports [30, 31]. Asian American women who had no usual source of care, no private insurance, were not married, were not acculturated, those who had poor health status, had infrequent visits to the doctors, and those who had low health literacy were consistently found to have lower mammogram uptake [32, 33, 34, 35]. In addition, mammogram uptake among Asian Americans was associated with fatalism [36], patient‐provider language and gender discordance [37], and ethnic identities [31]. In recent decades, breast cancer incidence has been rising among Asian American women [38, 39]. Moreover, the risk of breast cancer among younger foreign‐born Asian Americans was found to be higher than their US‐born counterparts [40]. This is a significant shift from the previous reports that found breast cancer risk was associated with length of stay in the United States, with younger Asian immigrants being at a lower risk [31]. The combination of low breast cancer screening adherence and rising breast cancer incidence in the Asian American community is alarming. If many Asian American women remain undiagnosed or delay breast cancer diagnosis until the cancer progresses to an advanced stage, they might experience invasive treatment procedures, and increased risk of mortality leading to a potential intergenerational impact due to maternal orphancy. Therefore, our findings call for urgent action to raise awareness of breast cancer risks and benefits of early detection in the Asian American community and to offer culturally tailored services in the clinics serving Asian Americans.

We found that women who were current smokers had lower odds of being adherent to breast cancer screening than those who never smoked, which is consistent with existing literature [41, 42]. However, this behavior could be a result of many factors. For example, active smokers have been found to have a lower uptake of preventative healthcare services (including screening mammograms) due to a lower perceived risk of smoking‐related diseases [43, 44, 45]. Other reports suggest that active smokers are aware of the health risks of smoking but avoid preventive care because they have higher fatalistic beliefs, fear of diagnosis, or fear of the need to quit smoking (when dealing with addiction) [46, 47]. Thus, interventions to raise awareness of the breast cancer risks and benefits of early detection among smokers are needed.

We also found that women with educational attainment below the tertiary level and those without health insurance had lower odds of adhering to mammogram uptake. Although mammogram programs exist for the uninsured through Medicare and other government‐funded programs, barriers associated with transportation [48], language [49], and health literacy [50] were found to deter mammogram uptake in these at‐risk populations. These barriers can be addressed through community‐based programs, such as *Friend to Friend*, which have been proven effective in increasing mammogram uptake [51, 52].

Being single/widowed/separated/divorced and having poor perceived health were inversely associated with breast cancer screening adherence in our study, which is consistent with the existing literature [53, 54]. Studies reported that women with poor perceived health conditions were more frequently non‐White, older, immigrants, of low socioeconomic status (based on income, employment, and education), and experiencing negative beliefs about breast cancer screening accessibility and acceptability [55], which could have resulted in low uptake in breast cancer screening.

In this study, women with a higher odds of being breast cancer screening adherent included NHBs, individuals with a personal cancer history, and those who have routine health checkups. Our finding of higher breast cancer screening among NHBs corroborates with previous studies [12, 13, 30]. This behavior could have resulted from a high perceived severity of breast cancer (due to high breast cancer–related mortality among the NHB population [56]), successful public health campaigns for NHBs on breast cancer prevention [57], and the creation of federal programs to increase access to mammograms for low‐income minorities, such as the *National Breast and Cervical Cancer Early Detection Program* [58].

Women with a personal cancer history were also found to have higher odds of breast cancer screening adherence. Previous studies found that the risk of secondary cancer or recurrence motivates cancer survivors to be adherent to breast cancer screening [59, 60]. However, variations in mammogram adherence exist within the survivor groups by sociodemographic characteristics [59, 61], knowledge [62], mastectomy history [63, 64], and the number of years that passed after treatment [63], which calls for targeted interventions to address the modifiable risk factors to increase equity in breast cancer screening adherence.

Women who had a routine health checkup in the past 2 years were found to be more adherent to breast cancer screening guidelines, which resonates with previous literature [65]. Moreover, the odds of breast cancer screening adherence for those who had a routine health checkup in the past 2 years were very high. This could be partially explained by the fact that respondents could have considered a mammogram to be part of routine health checkup. A routine checkup is a preventive healthcare service where, along with general healthcare checkups (blood pressure, cholesterol, and blood laboratory testing), healthcare professionals make cancer screening recommendations based on age, family history, and lifestyle [66]. In a study assessing physician priorities in the delivery of preventive care, annual routine checkups were found to be more time conducive to discussing preventive care than sick visits [67]. The physicians also added that breast cancer screening discussions in sick visits were of lower priority in comparison to smoking cessation, blood pressure, or lipid level control discussions [67]. This finding underscores the need to raise awareness of the benefits of routine physical examinations among women, which would help in the early detection of life‐threatening diseases like breast cancer. Due to a limited scope, our study was focused on assessing the association of routine checkups with breast cancer screening among US women. Future studies should examine disparities in routine checkup uptake and its association with cancer screenings in vulnerable populations such as tobacco users and minority racial groups.

Breast cancer screening adherence did not vary by sexual orientation in our study, which corroborates previous reports [68, 69]. Although this finding is reassuring, sexual orientation–related differences in breast cancer diagnosis and treatment have been reported in the literature [69]. Women of sexual and gender minorities are underrepresented in national surveillance, which makes this community less visible in disparity research and healthcare outreach.

This study had some limitations. Given the cross‐sectional design, a causal relationship between identified factors and adherence to breast cancer screening guidelines could not be established. Moreover, data were based on self‐report; hence, recall and social desirability bias could have affected women's responses. Nonetheless, the novelty of this research lies in the inclusion of vulnerable groups such as women who use tobacco, sexual minorities, and individuals with personal cancer history. Since the US population is heterogeneous, understanding the barriers and facilitators of various groups to effectively cater to the unmet needs associated with routine breast cancer screening and adherence is important to reduce breast cancer–related mortality in these groups.

## 5 Conclusion

Variations in breast cancer screening were observed across different demographic and behavioral subgroups. Asian race, current smoking, lower level of educational attainment, marital status (single/widowed/separated/divorced), uninsured status, and lower perceived health condition were found to be associated with lower breast cancer–screening adherence. Targeted interventions tailored for these vulnerable groups to raise awareness of the benefits of breast cancer screening are essential to increase surveillance and reduce the burden of breast cancer in the United States.

## Author Contributions

M.C. conceptualization, Methodology, Writing – original draft, Writing – review and editing. J.F.D. conceptualization, Methodology, Writing – original draft, Writing – review and editing. R.Y. data curation, Formal analysis, S.S. conceptualization, Data curation, Funding acquisition, Methodology, Project administration, Resources, Supervision, Writing – original draft, Writing – review and editing.

## Funding

The study was supported by the National Cancer Institute (P30CA016672 to S. Shete), the Betty B. Marcus Chair in Cancer Prevention (to S. Shete), the Duncan Family Institute for Cancer Prevention and Risk Assessment (S. Shete).

## Ethics Statement

The studies involving humans were approved by the Research Ethics Review Board of the National Center for Health Statistics. The studies were conducted in accordance with the local legislation and institutional requirements. The participants provided their written informed consent to participate in this study. The current study was exempted from ethics review because we used deidentified publicly available data.

## Conflicts of Interest

The authors declare no conflicts of interest.

## Supporting information


**Table S1:** Characteristics of the US age‐eligible female adult population stratified by breast cancer screening adherence—National Health Information Survey 2021.

## Data Availability

Publicly available datasets were analyzed in this study. This data can be found at: https://www.cdc.gov/nchs/nhis/documentation/2021‐nhis.html#cdc_data_surveillance_section_2‐using‐our‐data.
