# Supplementary material for: Adherence to Breast Cancer Screening Guidelines Among Age‐Eligible US Women: Findings From NHIS 2021
Source: Cancer Med. 2025 Dec 12;14(24):e71423. doi: 10.1002/cam4.71423 (PMC12700718; doi:10.1002/cam4.71423)
Supplement: Supplementary file 1 — Table S1: Characteristics of the US age‐eligible female adult population stratified by breast cancer screening adherence—National Health Information Survey 2021. [file CAM4-14-e71423-s001.docx]

**Table S1: Characteristics of the US age-eligible female adult population stratified by breast cancer screening adherence - National Health Information Survey 2021.**

| **Characteristics** | **Categories^@^** | **Overall** | |  | **Adherent** | | |  | **Not adherent** | | | | | | |  |  |
| --- | --- | --- | --- | --- | --- | --- | --- | --- | --- | --- | --- | --- | --- | --- | --- | --- | --- |
|  |  |  |  |  |  |  |  |  | **Never had a mammogram** | | |  | **Had a mammogram more than 2 years ago** | | |  |  |
|  |  | **N** | **wt. %** |  | **wt. %** | **95% CI^** | |  | **wt. %** | **95% CI** | |  | **wt. %** | **95% CI** | |  | **P value** |
|  |  | 6814 | 100 |  |  |  |  |  |  |  |  |  |  |  |  |  |  |
| Most recent mammogram | Within the past two years | 5200 | 76.4 |  |  |  |  |  |  |  |  |  |  |  |  |  |  |
|  | More than 2 years ago | 1302 | 18.7 |  |  |  |  |  |  |  |  |  |  |  |  |  |  |
|  | Never had a mammogram | 312 | 4.9 |  |  |  |  |  |  |  |  |  |  |  |  |  |  |
|  |  |  |  |  |  |  |  |  |  |  |  |  |  |  |  |  |  |
| Age (in years) | 50 - 64 | 4080 | 64.4 |  | 75.9 | 74.5 | 77.4 |  | 5.7 | 4.8 | 6.6 |  | 18.4 | 17.1 | 19.7 |  | 0.0038 |
|  | 65 - 74 | 2734 | 35.6 |  | 77.2 | 75.3 | 79.0 |  | 3.5 | 2.6 | 4.5 |  | 19.3 | 17.6 | 21 |  |  |
|  |  |  |  |  |  |  |  |  |  |  |  |  |  |  |  |  |  |
| Race or ethnicity | Non-Hispanic White | 4827 | 71.5 |  | 76.6 | 75.2 | 77.9 |  | 4.2 | 3.5 | 4.9 |  | 19.2 | 18.0 | 20.4 |  | <0.0001 |
|  | Non-Hispanic Black | 824 | 12.5 |  | 82.6 | 79.4 | 85.7 |  | 4.4 | 2.7 | 6.0 |  | 13.1 | 10.2 | 16.0 |  |  |
|  | Hispanic | 434 | 8.0 |  | 71.4 | 66.4 | 76.4 |  | 6.2 | 3.6 | 8.8 |  | 22.3 | 17.8 | 26.8 |  |  |
|  | Non-Hispanic Asian | 349 | 5.8 |  | 68.0 | 62.4 | 73.6 |  | 10.8 | 6.6 | 15.0 |  | 21.2 | 16.2 | 26.2 |  |  |
|  | Non-Hispanic others | 146 | 2.2 |  | 66.9 | 58.1 | 75.6 |  | 9.4 | 1.0 | 17.9 |  | 23.7 | 15.5 | 32.0 |  |  |
|  |  |  |  |  |  |  |  |  |  |  |  |  |  |  |  |  |  |
| Marital Status | Living as married or married | 3459 | 62.6 |  | 78.6 | 77.1 | 80.1 |  | 4.1 | 3.3 | 4.9 |  | 17.3 | 15.9 | 18.7 |  | <0.0001 |
|  | Single, widowed, separated, divorced | 3355 | 37.4 |  | 72.6 | 70.8 | 74.4 |  | 6.3 | 5.3 | 7.4 |  | 21.0 | 19.3 | 22.7 |  |  |
|  |  |  |  |  |  |  |  |  |  |  |  |  |  |  |  |  |  |
| Educational attainment | Less than High School | 571 | 11.6 |  | 64.9 | 60.1 | 69.8 |  | 12.0 | 8.4 | 15.6 |  | 23.0 | 19.0 | 27.0 |  | <0.0001 |
|  | High School/GED | 1634 | 33.2 |  | 73.5 | 71.1 | 75.9 |  | 6.3 | 4.9 | 7.7 |  | 20.2 | 18.1 | 22.4 |  |  |
|  | Some college | 735 | 11.9 |  | 81.1 | 77.8 | 84.4 |  | 3.9 | 2.3 | 5.5 |  | 15.0 | 12.2 | 17.9 |  |  |
|  | Bachelor’s and higher degree (tertiary) | 2515 | 43.3 |  | 81.0 | 79.3 | 82.7 |  | 2.8 | 2.1 | 3.6 |  | 16.2 | 14.6 | 17.7 |  |  |
|  |  |  |  |  |  |  |  |  |  |  |  |  |  |  |  |  |  |
| Poverty ratio^†^ | <2 | 381 | 5.0 |  | 67.4 | 61.5 | 73.3 |  | 11.0 | 6.4 | 15.6 |  | 21.6 | 16.8 | 26.3 |  | <0.0001 |
|  | ≥2 | 6433 | 95.0 |  | 76.8 | 75.6 | 78.1 |  | 4.6 | 4.0 | 5.3 |  | 18.5 | 17.4 | 19.6 |  |  |
|  |  |  |  |  |  |  |  |  |  |  |  |  |  |  |  |  |  |
| Urbanicity^#^ | Metropolitan | 5730 | 84.7 |  | 76.9 | 75.6 | 78.2 |  | 4.8 | 4.1 | 5.5 |  | 18.3 | 17.1 | 19.5 |  | 0.081 |
|  | Nonmetropolitan | 1084 | 15.3 |  | 73.3 | 70.8 | 75.9 |  | 5.8 | 4.0 | 7.6 |  | 20.9 | 18.5 | 23.2 |  |  |
|  |  |  |  |  |  |  |  |  |  |  |  |  |  |  |  |  |  |
| Born in US | Yes | 5611 | 79.4 |  | 76.8 | 75.6 | 78.1 |  | 4.5 | 3.8 | 5.1 |  | 18.7 | 17.5 | 19.9 |  | 0.0003 |
|  | No | 1014 | 17.4 |  | 72.9 | 69.7 | 76.2 |  | 7.2 | 5.3 | 9.1 |  | 19.9 | 17.0 | 22.7 |  |  |
|  | Missing | 189 | 3.1 |  | 83.9 | 77.1 | 90.6 |  | 4.3 | 1.0 | 7.7 |  | 11.8 | 6.1 | 17.5 |  |  |
|  |  |  |  |  |  |  |  |  |  |  |  |  |  |  |  |  |  |
| Health insurance | No | 308 | 5.3 |  | 43.4 | 36.8 | 50.0 |  | 22.2 | 16.9 | 27.5 |  | 34.4 | 28.3 | 40.5 |  | <0.0001 |
|  | Yes | 6492 | 94.7 |  | 78.3 | 77.1 | 79.4 |  | 4.0 | 3.4 | 4.6 |  | 17.8 | 16.7 | 18.9 |  |  |
|  |  |  |  |  |  |  |  |  |  |  |  |  |  |  |  |  |  |
| Routine checkup | Never or more than 2 years ago | 224 | 17.1 |  | 25.5 | 18.3 | 32.7 |  | 25.8 | 19.1 | 32.6 |  | 48.7 | 40.8 | 56.5 |  | <0.0001 |
|  | Checked less than 2 years ago | 1106 | 82.9 |  | 82.0 | 79.6 | 84.4 |  | 1.4 | 0.7 | 2.2 |  | 16.5 | 14.2 | 18.9 |  |  |
|  |  |  |  |  |  |  |  |  |  |  |  |  |  |  |  |  |  |
| Perceived health condition | Excellent/Very good/Good | 5555 | 81.5 |  | 78.0 | 76.7 | 79.3 |  | 4.7 | 4.0 | 5.4 |  | 17.3 | 16.1 | 18.4 |  | <0.0001 |
|  | Fair/Poor | 1257 | 18.5 |  | 69.1 | 66.0 | 72.3 |  | 5.9 | 4.4 | 7.5 |  | 25.0 | 22.0 | 27.9 |  |  |
|  |  |  |  |  |  |  |  |  |  |  |  |  |  |  |  |  |  |
| Personal cancer history | Yes | 1157 | 15.4 |  | 82.2 | 79.7 | 84.6 |  | 1.4 | 0.5 | 2.2 |  | 16.4 | 14.1 | 18.8 |  | <0.0001 |
|  | No | 5646 | 84.6 |  | 75.3 | 74.0 | 76.6 |  | 5.6 | 4.8 | 6.3 |  | 19.1 | 18.0 | 20.3 |  |  |
|  |  |  |  |  |  |  |  |  |  |  |  |  |  |  |  |  |  |
| Smoking status | Current smoker | 856 | 12.1 |  | 63.7 | 60.0 | 67.4 |  | 7.5 | 5.5 | 9.5 |  | 28.8 | 25.5 | 32.2 |  | <0.0001 |
|  | Former smoker | 1725 | 23.7 |  | 77.1 | 74.8 | 79.4 |  | 3.3 | 2.1 | 4.5 |  | 19.6 | 17.5 | 21.7 |  |  |
|  | Never smoker | 4056 | 61.3 |  | 78.1 | 76.7 | 79.6 |  | 5.2 | 4.3 | 6.1 |  | 16.7 | 15.4 | 18.0 |  |  |
|  | Unknown | 177 | 2.8 |  | 86.9 | 80.3 | 93.4 |  | 2.5 | 0.2 | 4.9 |  | 10.6 | 4.9 | 16.3 |  |  |
|  |  |  |  |  |  |  |  |  |  |  |  |  |  |  |  |  |  |
| Sexual orientation | Straight | 6355 | 96.9 |  | 76.4 | 75.2 | 77.6 |  | 5.0 | 4.3 | 5.6 |  | 18.6 | 17.5 | 19.7 |  | N/A |
|  | Gay or Lesbian | 101 | 1.4 |  | 77.9 | 69.3 | 86.5 |  | 1.8 | 0.0 | 4.0 |  | 20.3 | 11.9 | 28.7 |  |  |
|  | Bisexual | 49 | 0.6 |  | 61.7 | 47.6 | 75.7 |  | 7.0 | 0.0 | 14.8 |  | 31.4 | 17.3 | 45.4 |  |  |
|  | Something else | 25 | 0.4 |  | 83.8 | 70.0 | 97.6 |  | . | . | . |  | 16.2 | 2.4 | 30.0 |  |  |
|  | Don't know | 52 | 0.7 |  | 59.4 | 43.7 | 75.1 |  | 12.6 | 1.9 | 23.3 |  | 28.0 | 12.6 | 43.3 |  |  |
|  |  |  |  |  |  |  |  |  |  |  |  |  |  |  |  |  |  |

@ Some of the variable categories do not add up to 6814 because of missing responses.

^ Confidence interval

# Metropolitan includes geographical areas defined as large central metro, large fringe metro, medium, and small metro. Nonmetropolitan includes micropolitan and noncore.

† Ratio of family income to poverty threshold. The poverty threshold was obtained from the Census Bureau for the previous calendar year.
